# Natural history of coronary inflammatory activity in spontaneous coronary artery dissection: insights from serial pericoronary adipose tissue analysis

**DOI:** 10.1093/ehjcr/ytae115

**Published:** 2024-02-26

**Authors:** Satoshi Kitahara, Yu Kataoka, Yusuke Fujino

**Affiliations:** Department of Cardiology, Kashiwa Kousei General Hospital, 617 Shikoda, Kashiwa, Chiba, Japan; Department of Cardiovascular Medicine, National Cerebral and Cardiovascular Center, 6-1 Kishibe-shimmachi, Suita, Osaka 564-8565, Japan; Department of Cardiovascular Medicine, National Cerebral and Cardiovascular Center, 6-1 Kishibe-shimmachi, Suita, Osaka 564-8565, Japan; Department of Cardiology, Kashiwa Kousei General Hospital, 617 Shikoda, Kashiwa, Chiba, Japan

**Figure:**
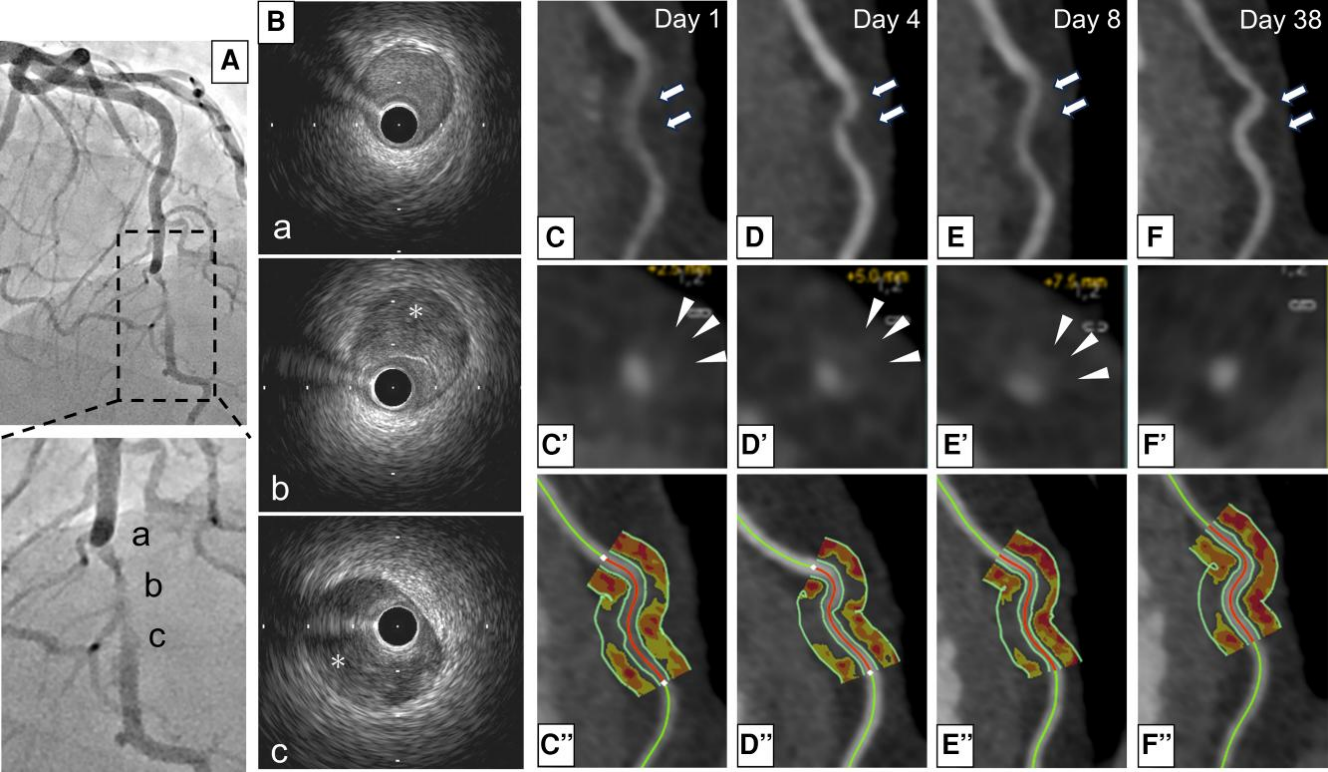


A 53-year-old woman was hospitalized due to ST-elevation myocardial infarction. Emergent coronary angiography revealed a severe stenotic lesion in her left anterior descending artery (*Panel A* and see Supplementary material online, *Video 1*). Intravascular ultrasound imaging revealed a narrowed true lumen compressed by intramural haematoma (Supplementary material online, *Video 2* and *Panel B*, a–c). She was diagnosed with spontaneous coronary artery dissection (SCAD). Following the commencement of medical therapies (aspirin 100 mg, bisoprolol 1.25 mg), this lesion was serially evaluated by coronary computed tomography angiography. At Day 1, diffuse lumen narrowing and wall thickening were observed. Furthermore, this lesion exhibited a high pericoronary adipose tissue (PCAT) attenuation [−73 Hounsfield unit (HU)], suggesting an elevated coronary inflammatory activity (*Panel C*). These features still continued to exist at Days 4 (−75 HU) and 8 (−78 HU) (*Panels D* and *E*). However, at Day 38, in addition to the disappearance of diffuse narrowing and wall thickening, PCAT attenuation improved (−91 HU) (*Panel F*).

Recent studies reported the association of SCAD with an increased perivascular inflammation. In our case, in addition to diffuse narrowing and wall thickening, high PCAT attenuation remained to exist by Day 8, and then these features improved at Day 38. Our observations show natural history of SCAD-related disease activity under medical therapies. Of note, continuing coronary inflammatory activity was observed by Day 8. This indicates a residual inflammation in the acute/sub-acute phase, which may be a potential substrate causing recurrence of SCAD. Further studies are required to elucidate whether evaluation of coronary inflammation could stratify a risk of recurrence in patients with SCAD.

## Supplementary Material

ytae115_Supplementary_Data

## Supplementary material

**Consent:** The authors confirm that written consent for submission and publication of this case report including the images and associated text has been obtained from the patient in line with COPE guidance.

**Data availability:** The data underlying this article will be shared on reasonable request to the corresponding author.

